# Comparison of Cleaning Efficacy and Instrumentation Time in Primary Molars: Mtwo Rotary Instruments vs. Hand K-Files

**DOI:** 10.7508/iej.2015.04.006

**Published:** 2015

**Authors:** Fatemeh Ramezanali, Farzaneh Afkhami, Ali Soleimani, Mohammad Javad Kharrazifard, Farshid Rafiee

**Affiliations:** a *Department of Endodontics, Dental School, Tehran University of Medical Sciences, International Campus, Tehran, Iran; *; b *Mofid Hospital, Shahid Beheshti University of Medical Sciences, Tehran, Iran****; ***; c *Statistical Consultant, Dental Research Center, Tehran University of Medical Sciences, Tehran, Iran****; ***; d *General Dentist, Tehran, Iran*

**Keywords:** Deciduous Tooth, Hand K-files, Mtwo, Primary Molars, Pulpectomy, Root Canal Preparation, Root Canal Therapy

## Abstract

**Introduction:**

Pulpectomy is the preferred treatment for restorable primary teeth with symptomatic irreversible pulpitis or periradicular lesion. Considering the rather new application of rotary files for pulpectomy of primary teeth, the aim of this study was to compare the cleaning efficacy and instrumentation time of hand K-files and Mtwo rotary system for preparation of human primary molars.

**Methods and Materials:**

This experimental study was conducted on 100 extracted primary maxillary and mandibular intact molars with no resorption. Access cavities were prepared and India ink was injected into the root canal on a vibrator using an insulin syringe. Canals were then divided into 5 groups (*n*=20): in group *I*, canals were instrumented using K-files up to #25 for mesial and buccal canals and #30 for palatal and distal canals. In group *II*, canals were prepared using Mtwo rotary files (15/0.05, 20/0.06 and 25/0.06 for mesial and buccal canals and 15/0.05, 20/0.06, 25/0.06 and finally 30/0.05 for distal and palatal canals). In group *III*, root canals were only irrigated with saline. Groups *IV* and *V* were the positive and negative control groups, respectively. The time required for cleaning and preparation of the canals for each of the specimens in groups *I, II* and *III* was recorded.

**Results:**

The mean score of cleanliness of Mtwo was not significantly different from K-file group (*P*>0.05). However the mean instrumentation time in Mtwo group was significantly shorter (*P*<0.001).

**Conclusion:**

Although there were no differences regarding the cleaning efficacy of either system, Mtwo rotary files were far more time efficient.

## Introduction

Pulpectomy is the treatment of choice for primary teeth with necrotic or irreversibly inflamed pulps [1]. Root canal preparation is the most time consuming step of root canal therapy (RCT), especially in pediatric dentistry [2]. The introduction of engine-driven files made of nickel titanium (NiTi) alloy was a major development in root canal shaping [3]. However, their application is dominantly restricted to permanent teeth [4]. Variable shapes and systems of engine-driven files are available in the market and Mtwo (VDW, Munich, Germany) is among the most commonly used systems [5]. Some advantages of Mtwo system are the ability to preserve the working length and canal curvature and better cutting efficacy [6].

Several studies have evaluated the cleaning efficacy of engine-driven and hand files on permanent molars; however, few studies have been conducted on primary molars [7]. Moghaddam *et al.* [8] compared the instrumentation time and cleaning efficacy of primary molars using hand files and FlexMaster rotary system. They found no significant difference in cleaning efficacy at middle and apical thirds, but the cervical third was more effectively cleaned with hand K-files. They showed that using FlexMaster system was less time consuming. 

In another study, Pinheiro *et al.* [7] compared the intracanal bacterial reduction and cleaning effectiveness of manual and rotary instrumentation techniques in deciduous molars. Their results revealed no significant differences in cleaning efficacy using hand K-files and ProTaper rotary instruments. However, manual instrumentation resulted in production of more smear layer. In contrast, Kummer *et al.* [9] reported less dentin removal after rotary instrumentation using Hero 642 system.

Schäfer *et al.* [10] compared the cleaning and shaping efficacy of ProTaper, RaCe and Mtwo rotary systems in preparation of severely curved root canals of extracted teeth. They concluded that Mtwo rotary instruments had superior cleaning efficacy; but no significant difference was noted regarding the smear layer removal ability in apical third of root canals [8]. Also Sonntag *et al.* [11] and Giovannone *et al.* [12] independently reported no significant differences in cleaning efficacy of Mtwo and ProTaper systems. 

Considering controversies and the popularity of rotary endodontic systems, this study aimed to compare the cleaning efficacy and instrumentation time of Mtwo and hand K-files in root canal preparation of primary molars.

## Materials and Methods

Sample size calculation was performed using two means option of the Minitab software. Considering *α*=0.05 and *β*=0.2, and Standard deviation of 1.2 (considering the result reported by Azar *et al.* [6]), the sample size was estimated 20 per group. A total of 100 extracted maxillary and mandibular primary first and second molars were immersed in 0.5% sodium hypochlorite solution for one week to eliminate organic debris. Then the teeth were transferred to saline solution. The selected teeth had sound crowns and roots with no anomaly, severe curvature, resorption, fracture or crack. 

For working length (WL) determination, #10 or #15 K-files (Mani Inc., Tochigi, Japan) were used depending on the canal diameter. The file was introduced into the canal until its tip was visible at the apex. WL was determined 1 mm short of the apex. Using an insulin syringe, India ink was injected into the canals on a vibrator except negative control group. Next, the teeth were stored in humid environment (wet gauze). To eliminate excess ink, the pulp chamber was cleaned with cotton pellets several times. Next, specimens were divided into 5 groups (*n*=20). In group *I*, the mandibular mesial and maxillary buccal canals were filed up to #25. Maxillary palatal and distal canals were filed up to #30 with hand K-files. After using each file, the canals were irrigated with saline. A chronometer (Darman Teb, Tehran, Iran) was used to record the instrumentation time. The time duration from the onset of filing to its termination (after using #25 or #30 K-file) was recorded. In all experimental groups, the time spent for canal irrigation and file exchange was also included. 

In group *II*, after canal negotiation with #10 or #15 hand K-files, Mtwo rotary instruments (VDW, Munich, Germany) were used: 15/0.05, 20/0.06 and 25/0.06 prepared the mesial and buccal canals and that were accompanied by additional 30/0.05 file for distal and palatal canals. In group *III*, irrigation of the root canals was done with 10 cc saline solution using a 23 gauge sterile syringe. Groups *IV* and *V* were the positive and negative control groups that were filled with ink and left untouched, respectively.

All preparations were done by the same operator. The access cavities were temporarily restored with Cavit and the teeth were immersed in saline solution. After cleaning and instrumentation, the canals were cleared as described by Silva *et al.* [13], to clearly observe the three-dimensional structure of the root canal and assess the cleaning efficacy of understudy methods. Briefly, the teeth were put individually in jars with a lid, containing 10% chloridric acid for 3 days. The liquid was renewed every day until the complete decalcification of the samples. Then the teeth were stored in running tap water for 8 h and dehydrated in 70% alcohol for 16 h and 90% alcohol for 3 h, that was refreshed hourly and finally the samples were kept in absolute alcohol for 3 h. After dehydration, the teeth were placed in methyl salicylate. 

Following the clearing steps, three blinded observers (endodontists and pedodontists) separately evaluated all specimens under a stereomicroscope (Motic K series, Motic Deutschland GmbH, Wetzlar, Germany) with 10× magnification. A four-grade scoring system was used for grading the cleaning efficacy of the root canal system (RCS), as described by Silva *et al.* [13]; *score 0*: complete ink removal (the canal was completely clean and no ink remained in any part of the root canal), *score 1*: almost complete ink removal (traces of ink found in some areas), *score 2*: partial ink removal (ink found on some walls in some areas larger than pinpoints or as interrupted short lines of ink less than 0.5 mm on the walls), and *score 3:* no ink removal (appreciable amount of ink, larger than 1 mm, were present on some areas of the canal walls). 

Data were analyzed using the ANOVA and Tukey’s HSD tests to compare the efficacy of cleaning between the two methods. In addition, the T-test was applied to compare the instrumentation time of hand and rotary files. The level of significance was set at 0.05.

## Results

No sign of ink penetration was seen in cervical, middle or apical thirds of root canals in group* V* (negative control group). In group *IV* (positive control group), ink had completely penetrated into the root canals. Comparison of the mean cleaning efficacy in the three experimental groups revealed significant differences in the cervical third of the root canals. As seen in Table 1, the most efficient cleaning was noticed at the cervical third of group *II* (Mtwo); while the lowest cleaning was in group *III* (saline) (*P*<0.001). The mean cleaning score for the middle third of the root canals was significantly different among the three experimental groups (*P*<0.001). Based on the results presented in Table 1, at the middle third of the root canals, the highest and lowest cleaning efficacy belonged to groups *II* and *III*, respectively. The mean cleaning efficacy was significantly different among the three experimental groups at the apical third of the root canal (*P*<0.001). The most and the least efficient cleaning was seen at the apical third of root canals in group *II *and *III.* The instrumentation time was significantly different among the groups *I*, *II* and *III*. Preparation in group *III* and group *I* was the fastest and the slowest, respectively (Table 2).

## Discussion

The present study compared the cleaning effectiveness and instrumentation time of manual and Mtwo rotary methods in preparation of human primary molars. According to the result of this study Mtwo rotary system allows more efficient root canal preparation, however this difference was not statistically significant.

Elimination of microorganisms from the root canal system (RCS) by appropriate cleaning and shaping is the most important factor ensuring the success of endodontic treatment. Thus, appropriate cleaning, shaping and sealing of the RCS are essential [1]. Endodontic instruments and techniques have undergone numerous modifications to achieve the highest cleaning and shaping efficacy. At present, NiTi engine-driven files are highly popular because they are efficient and safe especially for preparation of fine curved canals due to their high flexibility and elasticity [14, 15]. 

In this study, sound, extracted first and second primary molars were used. For the purpose of standardization, teeth with no anomaly, severe curve, resorption, fracture or crack were selected. Since the physiological age of individuals is not always related to their dental age, no chronologic limitation was set. During root canal preparation, irrigation with saline solution was done to eliminate the confounding effect of chemical irrigants on remaining debris. 

The ink penetration and clearing technique is useful to obtain information on various aspects of endodontic treatment including morphology of human teeth, studying the cleaning ability of the instrumentation and obtaining information on the quality of canal obturation [16]. The primary advantage of clearing method is its simplicity and rapid results that are revealed in few days, in comparison with other methods. This technique makes the teeth transparent; therefore the pulp space and canal walls become observable and the canal can be evaluated three-dimensionally [6, 16]. India ink, used in this study, remains stable during the experimental steps [17].

**Table 1:** The Mean±SD of cleaning score at the cervical, middle and apical thirds. Different superscribed symbols show significant differences (*P*<0.05)

**Group**	**Cervical third**	**Middle third**	**Apical third**
**Mtwo**	1.35±1.04^*^	1.15±0.93^*^	0.80±0.69^*^
**K-file**	2.20±0.52^*¥^	1.75±0.55^*^	1.00±0.72^*^
**Saline**	2.50±0.51^¥^	2.70±0.47^¥^	2.30±0.73^¥^

The results of the current study showed no significant differences in cleaning efficacy of Mtwo rotary system and hand K-files although the Mtwo rotary system showed higher efficacy in elimination of ink from the root canal walls. This finding was in agreement with the results reported by Silva *et al. *[13]. They evaluated the efficacy of ProFile rotary system and hand files in elimination of dye from the root canals of 33 primary teeth and demonstrated their similar efficacy in this regard. Another study reported better cleaning efficacy, especially in coronal and middle thirds, using ProTaper rotary and WaveOne systems compared to manual instrumentation which was in contrast to the results obtained in the present study [18]. Also, Crespo *et al.* [18] reported more favorable canal taper after instrumentation using ProFile 0.04 rotary files compared to hand K-files. Ahlquist *et al. *[19] compared the cleaning capacity of hand files and rotary instruments in root canals of permanent teeth and showed superior efficacy of hand filing for cleaning of the entire root canal length. They used ProFile (0.04 and 0.06) system in their study and also used sodium hypochlorite as intracanal irrigant. They longitudinally split the teeth and evaluated the penetration of dye into the tooth structure two-dimensionally. They used permanent teeth, which have fewer irregularities compared to primary teeth. In our study, the cleaning efficacy of both canal preparation systems was greater at the apical third compared to middle and coronal thirds of the root canals, which may be attributed to the site of entry of ink into the RCS. As explained earlier, India ink was injected by insulin syringe from the canal orifice into the RCS; thus, the concentration of ink was probably the highest at the coronal and then followed by middle thirds of the root canal. Foschi *et al*. [20] compared Mtwo and ProTaper rotary systems and reported that both rotary systems created a clean, debris-free surface at the coronal and middle thirds of the RCS. 

In the current study, in group *III*, after injection of India ink, only saline solution was used for root canal cleaning and no filing was done; the results showed lower cleaning scores in this group compared to the other experimental groups (rotary and hand files). However, compared to the positive control group, the difference in cleaning efficacy was not significant. In saline group, cleaning efficacy at the apical third was better than the middle and cervical thirds; which is probably due to the effect of solution injection via the canal orifice. 

**Table 2:** The Mean±SD of instrumentation time (sec) among the three groups. Different superscribed symbols show significant differences (*P*<0.05

**Group**	**Time (Sec)**
**Mtwo**	65.45±0.78^*^
**K-file**	131.05±0.95^¥^
**Saline **	8.30±0.24^€^

The current study showed that the instrumentation time for preparation of the primary molars with Mtwo rotary system (65 sec) was significantly shorter compared to the hand K-files (131 sec) (*P*<0.001). This finding is in agreement with the results reported by Rosa *et al.* [21], Ozen and Akgun [22] and Schäfer *et al.* [10] on permanent and Silva *et al.* [13] and Barr *et al. *[23] on primary teeth.

## Conclusion

The cleaning efficacy of Mtwo rotary instruments was the same as hand K-files in apical, middle and cervical thirds of primary molar root canals but the Mtwo system required shorter instrumentation time. Thus, Mtwo rotary system can be used as a suitable alternative for pulpectomy of deciduous molars.

## References

[1] Bowen JL, Mathu-Muju KR, Nash DA, Chance KB, Bush HM, Li H-F (2012). Pediatric and general dentists' attitudes toward pulp therapy for primary teeth. Pediatric Dent.

[2] Bhat C (2010). Sealing ability of newer generation bonding agents in primary teeth-an in vivo study. Int J Clin Den Sci.

[3] Alves RAA, Souza JB, Alencar AHG, Pécora JD, Estrela C (2013). Detection of procedural errors with stainless steel and NiTi instruments by undergraduate students using conventional radiograph and cone beam computed tomography. Iran Endod J.

[4] Lin C-P, Li U-M, Guo M-K (2006). Application of Ni-Ti rotary files for pulpectomy in primary molars. J Dent.

[5] de Lima Machado ME, Sapia LAB, Cai S, Martins GHR, Nabeshima CK (2010). Comparison of two rotary systems in root canal preparation regarding disinfection. J Endod.

[6] Azar M-R, Mokhtare M (2011). Rotary Mtwo system versus manual K-file instruments: Efficacy in preparing primary and permanent molar root canals. Indian J Dent Res.

[7] Pinheiro S, Araujo G, Bincelli I, Cunha R, Bueno C (2012). Evaluation of cleaning capacity and instrumentation time of manual, hybrid and rotary instrumentation techniques in primary molars. Int Endod J.

[8] Moghaddam KN, Mehran M, Zadeh HF (2009). Root canal cleaning efficacy of rotary and hand files instrumentation in primary molars. Iran Endod J.

[9] Kummer TR, Calvo MC, Cordeiro MMR, de Sousa Vieira R, de Carvalho Rocha MJ (2008). Ex vivo study of manual and rotary instrumentation techniques in human primary teeth. Oral Surg Oral Med Oral Pathol Oral Radiol Endod.

[10] Schäfer E, Vlassis M Comparative investigation of two rotary nickel–titanium instruments. ProTaper versus RaCe.

[11] (2004). Cleaning effectiveness and shaping ability in severely curved root canals of extracted teeth. Int Endod J.

[12] Sonntag D, Ott M, Kook K, Stachniss V (2007). Root canal preparation with the NiTi systems K3, Mtwo and ProTaper. Aust Endod J.

[13] Giovannone T, Migliau G, Bedini R, Ferrari M, Gallottini L (2008). Shaping outcomes using two Ni-Ti rotary instruments in simulated canals. Minerva Stomatol.

[14] Silva LA, Nelson-Filho P, Leonardo MR, Tanomaru JM (2004). Comparison of rotary and manual instrumentation techniques on cleaning capacity and instrumentation time in deciduous molars. J Dent Child (Chic).

[15] Gundappa M, Bansal R, Khoriya S, Mohan R (2014). Root canal centering ability of rotary cutting nickel titanium instruments: A meta-analysis. J Conserv Dent.

[16] O'Hoy P, Messer H, Palamara J (2003). The effect of cleaning procedures on fracture properties and corrosion of NiTi files. Int Endod J.

[17] Venturi M, Prati C, Capelli G, Falconi M, Breschi L (2003). A preliminary analysis of the morphology of lateral canals after root canal filling using a tooth-clearing. Int Endod J.

[18] Ojeda F, González M, Mariel J, Gutierrez F, Silva-Herzog D, Oliva R (2014). Evaluation of Lateral Condensation Endodontic Technique with 7/D11 Spreader, Finger Spreaders and An Ultrasonic Activated Tip. Europ Scientific J.

[19] Crespo S, Cortes O, Garcia C, Perez L (2008). Comparison between rotary and manual instrumentation in primary teeth. J Clin Pediatr Dent.

[20] Ahlquist M, Henningsson O, Hultenby K, Ohlin J (2001). The effectiveness of manual and rotary techniques in the cleaning of root canals: a scanning electron microscopy study. Int Endodontic J.

[21] Foschi F, Nucci C, Montebugnoli L, Marchionni S, Breschi L, Malagnino V, Prati C (2004). SEM evaluation of canal wall dentine following use of Mtwo and ProTaper NiTi rotary instruments. Int Endod J.

[22] Rosa FM, Modesto A, Faraco-Junior IM (3000). Manual and rotary instrumentation techniques for root canal preparation in primary molars. Dentistry.

[23] Ozen B, Akgun OM (2013). A Comparison of Ni-Ti Rotary and Hand Files Instrumentation in Primary Molars. J Int Dent Med Res.

[24] Barr ES, Kleier DJ, Barr NV (1998). Use of nickel-titanium rotary files for root canal preparation in primary teeth. Pediatric Dent.

